# P-1531. Endothelial and Macrophage Activation Biomarkers as Predictors of Severity and Clinical Outcomes in Severe Scrub Typhus

**DOI:** 10.1093/ofid/ofaf695.1712

**Published:** 2026-01-11

**Authors:** George M Varghese, Mukta Wyawahare, Navneet Sharma, Dhruva Chaudhary, Sanjay Mahajan, Kavitha Saravu, Ramya M, Nicholas PJ Day

**Affiliations:** Christian Medical College, Vellore, Tamil Nadu, India; JIPMER, Puducherry, Puducherry, India; Postgraduate Institute of Medical Education and Research, Chandigarh, Chandigarh, India; PGIMS, Rohtak, Haryana, India; IGMC, Shimla, Himachal Pradesh, India; Kasturba Medical College, Manipal, Karnataka, India, Manipal, Karnataka, India; Christian Medical College, Vellore, Vellore, Tamil Nadu, India; MORU, Bangkok, Krung Thep, Thailand

## Abstract

**Background:**

Scrub typhus is a zoonotic bacterial infection endemic to South and Southeast Asia, known for its potential to cause severe illness, including multi-organ dysfunction and death. Identifying biomarkers that can reliably predict disease severity and outcomes early during hospitalization is crucial for improving clinical management. This study investigated the prognostic value of biomarkers, ICAM-1, VCAM-1, sCD163, and IL-6, reflecting endothelial and macrophage activation in patients with severe scrub typhus

**Methods:**

A cohort of 670 patients with severe scrub typhus enrolled in the INTREST trial was studied. Plasma levels of ICAM-1, VCAM-1, sCD163, and IL-6 were measured on admission (day 0) and on day 7. Clinical data on organ dysfunction, ICU admission, need for ventilation or inotropic support, and mortality were recorded. Statistical analyses included Wilcoxon tests, logistic regression (adjusted for baseline), and multivariable models to evaluate associations between biomarker levels and disease severity and clinical outcomes, including 28-day mortality.

**Results:**

Biomarker levels were significantly higher in non-survivors and in patients with multiple organ involvement or requiring ICU care. At baseline, elevated ICAM-1, VCAM-1, sCD163, and IL-6 levels were associated with increased severity, including multiple organ involvement (p < 0.001). On day 7, levels of sCD163 and IL-6 remained significantly elevated in non-survivors. Multivariable analysis identified sCD163 as an independent predictor of severity and 28-day mortality (p = 0.001). Predictors of persistent complications included cardiovascular, respiratory, neurological, renal, and hepatic involvement.

**Conclusion:**

Elevated levels of endothelial (ICAM-1) and macrophage activation markers (sCD163, IL-6) are associated with increased severity and poor outcomes in severe scrub typhus. Persistent elevation by day 7 predicts mortality and complications, highlighting the potential of these biomarkers for early risk stratification and to guide critical care interventions in endemic settings.

**Disclosures:**

All Authors: No reported disclosures

